# Late treatment with imatinib mesylate ameliorates radiation-induced lung fibrosis in a mouse model

**DOI:** 10.1186/1748-717X-4-66

**Published:** 2009-12-21

**Authors:** Minglun Li, Amir Abdollahi, Hermann-Josef Gröne, Kenneth E Lipson, Claus Belka, Peter E Huber

**Affiliations:** 1 Department of Radiation Oncology German Cancer Research Center (DKFZ), Im Neuenheimer Feld 280, Heidelberg 69120, Germany; 2 Molecular Pathology, German Cancer Research Center (DKFZ), Im Neuenheimer Feld 280, Heidelberg 69120, Germany; 3 3M Pharmaceuticals, St. Paul, MN 55144, USA; 4 Department of Radiation Oncology, University of Heidelberg Medical School, Heidelberg 69120, Germany; 5 Department of Radiation Oncology, University of Munich medical school, Munich 81377, Germany; 6 Fibrogen, Inc., South San Francisco, CA 94080, USA

## Abstract

**Background:**

We have previously shown that small molecule PDGF receptor tyrosine kinase inhibitors (RTKI) can drastically attenuate radiation-induced pulmonary fibrosis if the drug administration starts at the time of radiation during acute inflammation with present but limited effects against acute inflammation. To rule out interactions of the drug with acute inflammation, we investigated here in an interventive trial if a later drug administration start at a time when the acute inflammation has subsided - has also beneficial antifibrotic effects.

**Methods:**

Whole thoraces of C57BL/6 mice were irradiated with 20 Gy and treated with the RTKI imatinib starting either 3 days after radiation (during acute inflammation) or two weeks after radiation (after the acute inflammation has subsided as demonstrated by leucocyte count). Lungs were monitored and analyzed by clinical, histological and *in vivo *non-invasive computed tomography as a quantitative measure for lung density and lung fibrosis.

**Results:**

Irradiation induced severe lung fibrosis resulting in markedly reduced mouse survival *vs*. non-irradiated controls. Both early start of imatinib treatment during inflammation and late imatinib start markedly attenuated the development of pulmonary fibrosis as demonstrated by clinical, histological and qualitative and quantitative computed tomography results such as reduced lung density. Both administration schedules resulted in prolonged lifespans. The earlier drug treatment start resulted in slightly stronger beneficial antifibrotic effects along all measured endpoints than the later start.

**Conclusions:**

Our findings show that imatinib, even when administered after the acute inflammation has subsided, attenuates radiation-induced lung fibrosis in mice. Our data also indicate that the fibrotic fate is not only determined by the early inflammatory events but rather a complex process in which secondary events at later time points are important. Because of the clinical availability of imatinib or similar compounds, a meaningful attenuation of radiation-induced lung fibrosis in patients seems possible.

## Background

Radiotherapy is a mainstay of treating neoplasm in lungs [1-4]. Fibrosis is the main chronic side effect of radiotherapy that potentially prevents to deliver the necessary dose to benefit cancer patients [5-7]. Although modern techniques of radiotherapy (stereotactic radiotherapy, intraoperative radiotherapy, interstitial brachytherapy etc.) are now increasingly used to improve dose distribution and reduce side effects, radiation induced fibrotic lesions still occur [8-10]. There has been remarkably little progress in the development of effective antifibrotic therapies [7,11].

Recent studies indicated that pulmonary fibrosis is not a unique pathologic process but rather an excess of the same biologic events involved in normal tissue repair with persistent and exaggerated wound healing ultimately leading to an excess of fibroblast replication and matrix deposition [7,11]. A cascade of many cytokines leading to lung fibrosis after radiation injury has been described [12,13]. Recently, a number of new regulators in radiation-induced lung injury such as intercellular adhesion molecules (ICAM-1) and the CD95 ligand system have been reported [14,15]. Typical fibrogenic mediators include TGF-β, IL-1, TNF-α, bFGF, and thrombin, but also PDGF has been implicated to demonstrate profibrotic activities [16-21]. Thus the PDGF/PDGFR system can be considered as a promising target for treating fibrotic diseases [22-26].

Recently, we have shown that PDGF receptor tyrosine kinase inhibitors (RTKI) including imatinib can attenuate radiation-induced pulmonary fibrosis if the drug administration starts before the toxic event or within three days after the insult [27]. PDGF RTKI prolonged survival and protected mice from lung fibrosis, presented as reduced lung density measured by computed tomography examinations, although the radiation-induced acute inflammation was not significantly abrogated. Thus we hypothesized that fibrogenesis is a separate process after acute inflammation, correlated but not dependent to acute inflammation [27].

However in this previous study PDGF RTKI (SU9518) was administrated during RT-induced acute inflammation (1 day before and 3 days after radiation) showing no marked but certain effects on the acute inflammation. Therefore, acute inflammation is affected to some extent by concurrent drug administration and, even if the measurable extent of inflammation had not been significantly affected, the administration during inflammation could still be a prerequisite for the later antifibrotic effects. Thus, in the present study we chose a late drug treatment starting at the time when the primary inflammation has subsided, to rule out direct and indirect effects associated with acute inflammation,

Therefore we report here the full results of the imatinib experiments including the data on the late imatinib administration arm starting two weeks after radiation, at a time when the acute inflammation has already completely subsided. These experiments thus investigate i) the role of acute inflammation in the development of radiation induced lung fibrosis, ii) if attenuation of the acute inflammation is necessary to block fibrosis and iii) if a late drug administration after radiation insult and after the acute inflammation has subside still has antifibrotic potential. To this end thorax of C57BL/6 mice were irradiated with 20 Gy and mice were subsequently treated with imatinib mesylate/Gleevec starting either three days after radiation or two weeks after radiation. Longitudinal follow-up of the mice lungs *in vivo *was performed by noninvasive radiological monitoring using high resolution computed-tomography [28].

## Methods

### Experimental protocol and animal model

All animal procedures were approved by institutional and governmental authorities (Regierungspraesidium Karlsruhe, Germany). Fibrosis-prone mice (female C57BL/6J, 8 weeks old, approximate body weight 20 g, Charles River Laboratories, Sulzfeld, Germany) were used. For thoracic irradiation, mice were anesthetized by intraperitoneal application of Domitor (Pfizer, Exton, USA; 0.2 mg/kg) and Ketamin 10% (Park, Davis & Company, Berlin, Germany; 100 mg/kg). Cobalt-60 gamma radiation (Siemens Gammatron S, Erlangen, Germany) was administered as single dose (20 Gy) to the entire thorax (0.441 Gy/min; source surface distance: 0.7 m) using one standing field anterior-posterior. Other organs, above and beyond the thorax were shielded. Animals were supplied with diet and water *ad libitum*.

Imatinib Mesylate was provided by SUGEN Inc. South San Francisco, CA. To achieve clinically relevant doses [26,27], Imatinib were formulated in standard mouse chow at 0.5 mg/g resulting in a dosage of 40 mg/kg/d. Imatinib (Gleevec) is known to have high activity against three kinases: Bcr/Abl, c-Kit, and PDGFR-α and -β [26,27]. In irradiated animals, imatinib treatment was either started three days after radiation or two weeks after radiation and was continued until the end of observation, as stated in our previous study [27]. Animals were checked three times weekly, clinically examined and weighed.

### Lung histology

Histological analysis from mice tissues was performed systematically at early and later time points after radiation as described [27,28]. Briefly, lungs were fixed by intratracheal instillation of 4% formalin, followed by overnight fixation, embedding in paraffin, sectioned at 5 μm, and stained with hematoxylin-eosin (H&E). The total count of leukocytes was determined by morphometric evaluation (Q 600 Quantimet, Leica Microsystems, Wetzlar, Germany) and the septal thickness was measured in 5 regions of interest (ROI) for each mouse.

### High-resolution computed tomography (HRCT) of mouse lungs

To obtain an independent qualitative and quantitative measure for lung fibrosis in the mice we used high-resolution computed tomography (CT). CT is the method of choice for monitoring fibrosis in patients. This radiological method allows non-invasive and repeated measurements in the same mice in a longitudinal way [28]. CT exams were performed in 5 randomly selected mice from each group every second week during the entire observation period. CT images were captured on a Toshiba multi-slice CT scanner (Aquilion 32). 120 kV with 100 mAS were applied. 0.5 mm thin slices with 0.5 mm inter-slice distance spanned the complete mouse chest (total acquisition time 0.5 seconds). Multiplanar reconstructions (MPR) were performed for semiquantitative analysis. Hounsfield units (HU) of section slides from the upper and lower lung region were determined. Eight regions of interest (ROI) were defined in the following areas: the right upper anterior and posterior regions, the left upper anterior and posterior regions, the right lower anterior and posterior regions and the left lower anterior and posterior regions. Total arithmetic means ± SE of the HU were calculated.

### Statistics

Mouse survival curves after thoracic irradiation and imatinib treatments were calculated with the Kaplan-Meier method and compared using the log-rank test. Other quantitative data are given as mean values ± SD or as indicated. For analysis of differences between the groups, ANOVA followed by the appropriate post hoc test for individual comparisons between the groups was performed. All tests were two-tailed. *P *< 0.05 was considered statistically significant.

## Results

### Late imatinib interventive treatment prolongs mouse survival

**Figure 1:**
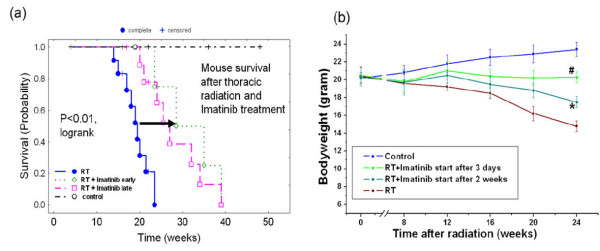
**(a) Kaplan-Meier analysis of mouse survival following thoracic irradiation and imatinib treatment**. Death was considered complete (cause-specific due to radiation) in all cases except those of planned euthanasia for histological assessment, which were considered as censored. Radiation (20 Gy) reduced survival (P < 0.001 vs. the control as reported in (24)). Imatinib treatment increased mouse survival if administration started as late as 2 weeks after radiation (P < 0.02 vs. radiation) and if started early within 3 days after radiation (P < 0.01 as reported in (24)). The earlier start of drug treatment tended to be more effective in prolonging survival than later start of drug treatment, but this difference was not significant (P > 0.1). **(b) **Bodyweight follow-up after thoracic irradiation and imatinib treatment. Five mice were randomly selected in each group and weighed every two weeks. Mean ± SE was presented. * P < 0.01 *vs*. the RT only group; # P < 0.01 *vs*. the control group.

A single dose of 20 Gy thoracic irradiation induced marked lung fibrosis and dramatically reduced animal survival versus nonirradiated animals. Median survival was 19 weeks after radiation *vs*. control mice which stayed alive for more than one year (*P *< 0.0001). The Kaplan-Meier curves are depicted in figure 1a. Imatinib treatment starting 3 d after radiation prolonged median survival by ~11 weeks with a median survival of 30 weeks (*P *< 0.01 vs. radiation alone) as shown in a previous study [27]. Importantly to us, imatinib treatment starting 2 weeks after radiation also prolonged median survival by ~8 weeks with a median survival of 27 weeks (*P *< 0.02 vs. radiation alone). The difference in survival between earlier and later treatment schedules was not statistically significant (P > 0.1), but a tendency was present suggesting that the earlier drug treatment start was beneficial.

To support the survival data we also analyzed the animals' clinical status. We found that imatinib treatment in both early and late treatment arms also attenuated radiation-related clinical adverse effects such as weight loss (figure 1b, P < 0.02, at all time points after week 14) and other clinical parameters, which were monitored weekly over the entire observation period. In specific, imatinib early and late improved clinical status including animal behavior (worse after irradiation, improved by imatinib), tachypnoea and heart rate (both higher after irradiation, reduced by imatinib). Again, a marked difference of the benefits caused by imatinib in the clinical animal parameters between the two imatinib schedules was not observed.

### Computed tomography of mice lungs

**Figure 2:**
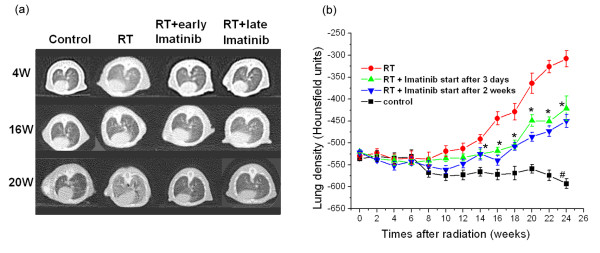
**(a) High resolution computed tomography (CT) as a non-invasive tool for qualitative and quantitative longitudinal monitoring of pulmonary fibrosis progression in mice**. Representative CT scans showing progression of pulmonary fibrosis in mice after 20 Gy whole thorax irradiation (RT), and treatment with imatinib treatment starting 3 days and 2 weeks after radiation (RT). Fibrosis is characterized by diffuse bilateral areas of "ground-glass" attenuation and intralobular reticular opacities. **(b) **Quantitative lung density values derived from CT scans. The same 5 randomly chosen mice in each treatment group were examined in a longitudinal study by CT every 2 weeks. 8 regions of interest (ROI) were randomly selected in the lungs and the lung density (in Hounsfield Units (HU)) was determined for each ROI. Mean ± SE are presented. * P < 0.01 *vs*. the RT only group; # P < 0.01 *vs*. the control group.

Computed tomography (CT) was used to obtain an independent qualitative and quantitative measure of mice lung fibrosis that could be repeated in the same animal over time. As reported before [24], after week 16 typical radiological features of lung fibrosis were visible after 20 Gy irradiation including irregular septal thickening, patchy peripheral reticular abnormalities with intralobular linear opacities and subpleural honeycombing (figure 2a). The extent of fibrotic disease progression in CT images correlated well with histology and clinical impairment. Imatinib treatment was able to markedly reduce the radiological/morphological signs of fibrosis after radiation in both early and late imatinib treated mice. In addition to the morphological assessment, CT enabled quantitation of fibrosis by an assessment of the lung density (quantified in Hounsfield units (HU)). We found that lung density drastically increased during weeks 12 to 24 post radiotherapy (figure 2b) in irradiated mice only. Imatinib in both early and late application arms strongly inhibited this increase by approximately 50% (P < 0.001). The earlier therapy start appeared to be slightly more effective in reducing CT signs of lung fibrosis than a later therapy, but this tendency did not reach statistical significance (P > 0.1).

### Histological assessment of lung fibrosis after irradiation

It is assumed that exposure of normal lung tissue to irradiation has two well-recognized adverse effects: acute/subacute pneumonitis and fibrosis as long term sequelae [11,27-29]. To better understand the pathogenesis of the radiation-induced lung fibrosis process, and to evaluate the modulation after radiation and imatinib, mice were selected for analysis of leukocyte infiltration, edema and collagen-deposition with associated thickening of the alveolar septum.

**Figure 3:**
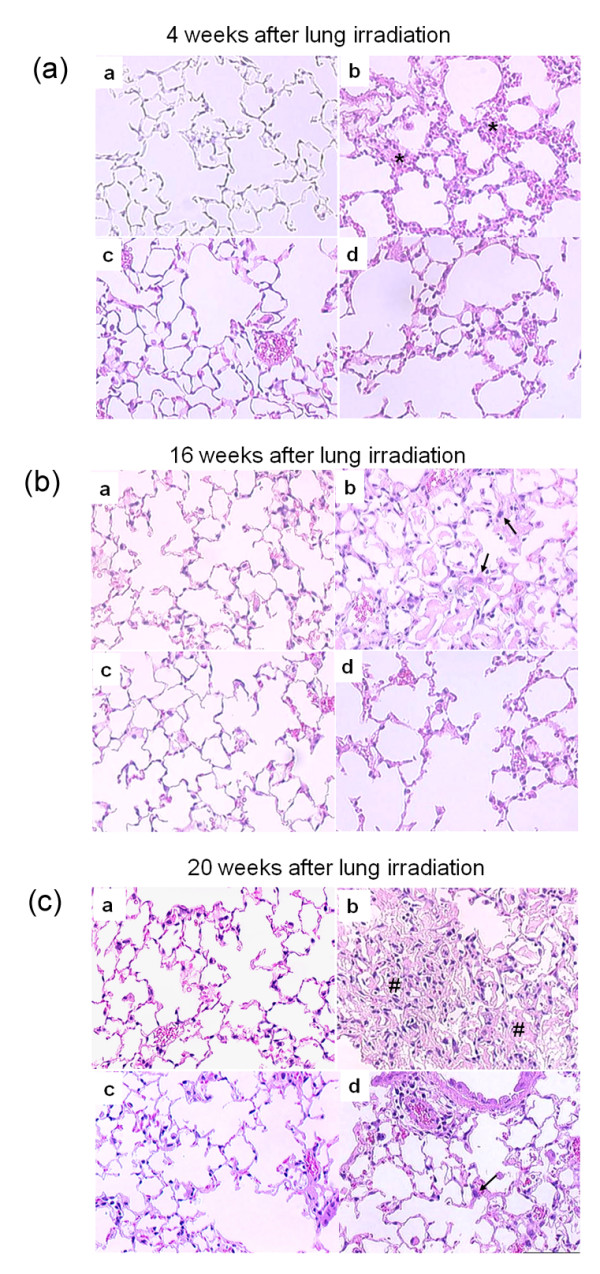
**(a) Photomicrographs of H&E stained mouse lung tissue sections from a) control mice, b) irradiated mice (20 Gy, RT) and mice treated with imatinib starting 3 days (c) or 2 weeks (d) after thoracic irradiation**. Leukocytes infiltration was marked with asterisk. **(b) **Photomicrographs of H&E stained mouse lung tissue sections at 16 weeks from a) control mice, b) irradiated mice (20 Gy, RT) and mice treated with imatinib starting 3 days (c) or 2 weeks (d) after thoracic irradiation. Fibroblasts were marked with arrow. **(c) **Photomicrographs of H&E stained lung tissue sections at 20 weeks from a) control mice, b) irradiated mice (20 Gy, RT) and mice treated with imatinib starting 3 days (c) or 2 weeks (d) after thoracic irradiation. Collagen depositions were marked with #.

**Figure 4:**
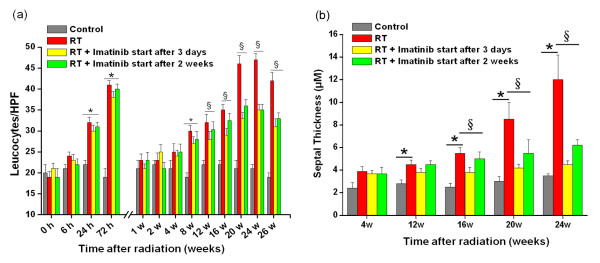
**(a) Quantitative analysis of leukocyte numbers as inflammation parameter**. Bars are mean ± SE. * p < 0.05 *vs*. controls. §p < 0.05 *vs*. radiation only for both early and late imatinib schedules. **(b) **Quantitative analysis of septal thickness as fibrotic parameter presenting deposition of extracellular collagen. Bars are mean ± SE. * p < 0.05 *vs*. controls. §p < 0.05 *vs*. radiation only, for both early and late imatinib schedules.

As described earlier a biphasic radiation response was observed, initially consisting of acute and subacute pneumonitis, which was followed by the onset of fibrogenesis [27]. The characteristic histologic findings in the pneumonitis phase of the radiation response were prominent inflammatory cell infiltrates in the alveoli and lung interstitium with simultaneous interstitial edema (figure 3a). Both parameters exhibited similar kinetics in the acute phase, reaching their maxima about 72 hours after radiation injury. After the acute radiation response, leukocyte count spontaneously subsided within one week (figure 4a). When imatinib administration started early during inflammation the treatment did not markedly decrease this first, radiation-induced acute leukocyte peak (P > 0.2), although imatinib affected the inflammation to some extent and, the tendency was seen that imatinib had a nonsignificant tendency to reduce acute inflammation in terms of reduced edema and leucocyte count.

Histological analysis of irradiated lungs further showed the development of fibrosis by progressive collagen deposition after week 12 (figure 3b). This fibrogenesis phase was characterized by development of typical fibroblast foci and exuberant deposition of extracellular matrix in irradiated lungs (figure 3c). Both imatinib schedules reduced collagen deposition and septal thickness (figure 4b), while the early administration appeared to be slightly more effective than late administration. In irradiated mice, the later fibrogenesis phase was accompanied by a strong second onset of leukocyte infiltration that began several weeks after irradiation and reached a peak at approximately 20 weeks post irradiation.

At later time points (> 20 weeks) the fibrotic foci evolved and coalesced into widespread fibrosis with remodeling of the lung architecture. Moreover, in the irradiated lungs the second onset of progressive fibrosis-related leukocyte infiltration persisted until the morphologically described fibrosis process was completed (after week 26). Figure 4a also shows that this second inflammatory response was also reduced in terms of reduced leucocyte count by both early and late imatinib treatment (P < 0.05). Here again, the earlier drug treatment start tended to be slightly more effective than the later treatment start, but this difference was not statistically significant (P > 0.5).

## Discussion

Here we confirm that imatinib (Gleevec^©^) treatment is an effective strategy to attenuate radiation-induced lung fibrosis in mice. The beneficial drug effects are present even when drug administration starts two weeks after radiation when the acute radiation associated inflammation is completely subsided. The antifibrotic drug effectivity after the radiation-induced inflammation suggests that the fibrotic fate after radiation is not completely determined by the early inflammatory events, but rather by complex secondary signalling processes [27-31]. Therefore the present paper confirms and extends previous publications on imatinib showing beneficial antifibrotic effects in a radiation induced lung fibrosis model if the drug treatment starts before or after the radiation insult but within the acute inflammation [27].

Both early and late drug treatment start were able to attenuate the development of radiation induced lung fibrosis as shown by histological analysis during the relative long time course of lung fibrogenesis of up to 26 weeks. Imatinib markedly attenuated the development of fibroblast foci and the subsequent remodeling of the lung architecture. The morphological beneficial effects of imatinib were in agreement with qualitative and quantitative high-resolution computed tomography scans of mouse lungs. Moreover, a significant survival benefit and reduced clinical morbidity in imatinib treated mice was seen for both treatment schedules.

When comparing the earlier (3 days) *vs*. the later imatinib treatment start (2 weeks after radiation) we found that the earlier therapy start was beneficial with respect to all endpoints tested (histology, survival, CT monitoring, clinical behaviour), but this advantageous tendency for early treatment did not reach statistical significance.

One assumption of the inflammation and fibrosis debates were that fibrosis could be avoided if the early inflammation cascade was interrupted before irreversible tissue injury occurred [7]. However, early anti-inflammatory therapies, even in combination with potent immunosuppressives, fail to improve the disease outcome in the clinical setting [7,11]. Therefore, acute inflammation is probably not the only critical step in the development of the fibrotic response. In our setting, although neither the early imatinib start markedly reduced the acute inflammatory response nor the later start did (which was scheduled on purpose to not interfere with acute inflammation), the drug still attenuated the onset and development of lung fibrosis. Conversely, the second inflammatory response occurring around 12 weeks and later after radiation, was dramatically attenuated by both imatinib schedules. Thus the inhibition of the later fibrogenesis consisting of stromal cell migration, proliferation and extracellular matrix deposition seems to be a principal drug target.

A viable hypothesis is that imatinib's antifibrotic effects in the radiation lung model are conveyed via inhibition of PDGF signalling, although imatinib has been demonstrated to show marked activity against at least three kinases: Bcr/Abl, c-Kit, PDGFR-α and -β which can all be linked to fibrosis, in particular in conjunction with TGF-β signalling [22-24]. The potential role of PDGF signalling for the development of lung fibrosis, and in turn for the treatment of fibrosis by inhibiting PDGF signalling is supported by data in idiopathic pulmonary fibrosis, asbestos-, bleomycin- and radiation-induced lung fibrosis as well as in fibrosis in other organs such as the kidneys, liver, skin and heart [16-18,23-26]. Therefore, together with data on radiation induced PDGF expression and phosphorylation of PDGFR *in vitro *and *in vivo*, and the inhibition by the kinase inhibitors, we believe that the inhibition of PDGFR signalling is a key mechanism behind our functional findings [27,30].

Nevertheless, one cannot exclude important primary roles of Bcr/Abl, c-Kit, or TGF-beta pathways and one should also keep in mind that ATP-competitive kinase inhibitors rarely exhibit complete selectivity. Therefore many additional data towards a better mechanistic understanding of our data could be obtained. At the same time, it will be difficult to proof that one or more specific kinase/kinases resulting in one or several protein expression event and no other cascade is responsible for the beneficial role of imatinib here. For example, although others and we had previously shown that PDGF RTKI inhibited phosphorylation of PDGFR *in vivo *and this likely contributed to the benefits, it has also been reported that imatinib's c-kit effects and its link to TGF-beta might be the responsible beneficial antifibrotic pathway [22].

Moreover, the data should also be interpreted with the understanding that there may also be additional potential off-target effects. Such off-target effects may have well contributed to the antifibrotic effects that these compounds have. Rather than pointing out a single protein or gene, it is conceivable that the development of fibrosis is not a single step event, but rather an imbalance of an otherwise physiological homeostatic system with many players involved. In these terms fibrogenesis may be described as a shift of the homeostatic system towards the profibrotic state with the consequence that the entire process can only be understood as a gene and protein network shift which may call for systematic biology approaches for a deeper and more correct understanding [32-34].

The network idea is perhaps fostered by the fact that e.g. PDGF signalling alone is not an exclusive feature of fibrosis research but also known as a key signalling in cancer research, since PDGF signalling is considered to be a driving force for cancer cells and known to be proangiogenic [35]. Accordingly, the inhibition of PDGF signalling is being investigated as anticancer drugs alone and in combination with chemotherapy and radiotherapy [36-40]. Therefore, a two-fold rationale for the use of PDGF RTKI in radiation oncology might unfold: first, employing the anticancer effects of PDGF inhibition while second, simultaneously decreasing fibrosis as a common adverse side effect in radiotherapy [41,42]. However, again, it is unlikely that single pathway inhibition can completely prevent lung fibrosis, considering the intricate genetic networking associated with this complex process. While our data indicate that fibrosis can be attenuated or delayed, it still progressed despite imatinib. Therefore, RTKI should be considered in the context of other drug therapies, especially since, as for any other drug, potential side effects e.g. cardiotoxicity have been reported for imatinib [43].

## Conclusions

Taken together, we demonstrate here that drug treatment using imatinib might be a useful therapeutic approach to attenuate radiation-induced lung fibrosis or other types of fibrosis, which exhibits benefits even after the damaging insult and its acute inflammation has completely subsided.

## Competing interests

The authors declare that they have no competing interests.

## Authors' contributions

ML performed experiments, analyzed data and participated in writing the manuscript. AA participated in designing the study and analyzed data. KEL participated in the study design and manuscript writing. HJG performed and analyzed histology. PEH designed the study, analyzed data, and wrote the manuscript. All authors approved the final version of the manuscript.
